# Myopericarditis Presenting With McConnell's Sign: A Case Report and Literature Review

**DOI:** 10.7759/cureus.109436

**Published:** 2026-05-22

**Authors:** Graham Rector, Mohammad F Bilal, Ronald Maag

**Affiliations:** 1 Cardiology, Baylor College of Medicine, Houston, USA; 2 Cardiology, The Texas Heart Institute at Baylor College of Medicine, Houston, USA

**Keywords:** mcconnell’s sign, myocarditis, myopericarditis, pericarditis, pulmonary embolism

## Abstract

McConnell’s sign is a classic echocardiographic finding most often encountered in the context of acute pulmonary embolism and is defined by severe hypokinesis or akinesis of the right ventricular free wall and hypercontractility of the apex. Despite having a high specificity for pulmonary embolism, other causes of McConnell’s sign have been reported. This case is a rare presentation of McConnell's sign in association with myopericarditis. The patient presented with a systemic lupus erythematosus flare as well as acute pleuritic chest pain and was found to have significantly elevated high-sensitivity troponin, pericardial effusion, and McConnell’s sign on echocardiography. Pulmonary embolism and underlying pulmonary hypertension were ruled out. The patient improved clinically with treatment of a systemic lupus erythematosus flare, and short-term follow-up echocardiography demonstrated resolution of right ventricular dysfunction. This case challenges the conventional diagnostic specificity attributed to McConnell’s sign for pulmonary embolism by describing a similar pattern of regional right ventricular wall motion abnormalities in the setting of an inflammatory myopericardial syndrome. Additionally, it underscores the utility of speckle-tracking strain analysis to help differentiate potential etiologies in right ventricular dysfunction. This case demonstrates the importance of having a broad differential when encountering McConnell’s sign and represents an underappreciated cause of McConnell’s sign associated with myopericarditis.

## Introduction

McConnell’s sign is an echocardiographic finding first described as a marker for acute pulmonary embolism (PE). The finding was first described by McConnell et al. in 1996 and is defined by severe hypokinesis or akinesis of the right ventricular (RV) free wall and hypercontractility of the RV apex [1]. These abnormalities are best appreciated on the standard echocardiographic windows used for RV chamber quantification, including the RV-focused apical four-chamber and subcostal views. Despite a relatively high specificity for acute PE of 97% [2], other causes have been reported in the literature. The mechanism underlying McConnell’s sign is not completely understood but is thought to be a combination of RV ischemia from increased wall stress and spherical force distribution leading to free wall bulging [1,3]. The apparent hypercontractility of the RV apex is understood to be a visual phenomenon due to tethering from a hyperdynamic left ventricle.

We offer a case of a patient who presented with an overlap of pericarditis and myocarditis syndromes and McConnell’s sign on echocardiography, which is an underappreciated and not well-described cause. This article reviews the latest American College of Cardiology (ACC) and European Society of Cardiology (ESC) guidelines for pericarditis and myocarditis, alongside typical RV echocardiographic findings. Furthermore, it examines the underlying pathophysiology of McConnell’s sign and the diagnostic patterns of myocarditis with RV involvement, emphasizing the necessity of a broad differential diagnosis in clinical practice.

## Case presentation

A 37-year-old female with a prior history of systemic lupus erythematosus (SLE) on hydroxychloroquine presented with subacute upper respiratory symptoms and a one-day episode of left-sided pleuritic chest pain, chills, and joint swelling.

Initial vital signs were a temperature of 97.9 degrees Fahrenheit, a blood pressure of 160/103 mmHg, a heart rate of 107 beats/min, a respiratory rate of 18 breaths/min, and an oxygen saturation of 100% on room air by pulse oximetry. On examination, they appeared in mild distress and with swollen and tender metacarpophalangeal and proximal interphalangeal joints on both hands. Cardiovascular examination revealed a regular tachycardia with a friction rub. Peripheral pulses were strong and symmetrical. Jugular venous pulse was not elevated, and there was no peripheral edema. Lungs were clear and without rales, rhonchi, or wheezes.

Laboratory evaluation showed a normal metabolic panel and complete blood count. The initial high-sensitivity troponin I was <4 pg/mL; however, on repeat testing, it was found to be 2,460 pg/mL and peaked at 2,535 pg/mL on day two of admission. NT-proBNP was normal at 51 pg/mL. D-dimer and CRP were elevated. Complements (C3 and C4) were low, and the double-stranded DNA IgG titer was elevated (Table 1).

**Table 1:** Laboratory findings on admission WBC: White blood cell; BUN: blood urea nitrogen; HS: high sensitivity; NT: N-terminal; BNP: brain natriuretic peptide; DsDNA: double-stranded deoxyribonucleic acid; CRP: C-reactive protein

Laboratory Test	Result	Normal Reference Range
WBC (per mm^3^)	3900	3500–1050
Hemoglobin (g/dL)	4.3	11.2–15.7
Hematocrit (%)	38.2	34.1–44.9
Platelets (per mm^3^)	284,000	150,000–450,000
Sodium (mEq/L)	145	136–145
Potassium (mEq/L)	4.1	3.4–5.1
Bicarbonate (mEq/L)	23	22–29
BUN (g/dL)	10	7–19
Creatinine (mg/dL)	0.85	0.50–1.00
Glucose (mg/dL)	130	70–105
HS Troponin I, Admission (pg/mL)	< 4	4–51
HS Troponin I, 24 hours (pg/mL)	2450	4–51
HS Troponin I, 30 hours (pg/mL)	2535	4–51
HS Troponin I, 36 hours (pg/mL)	1360	4–51
NT-proBNP (pg/mL)	51	0–125
D-Dimer (ng/mL)	1,466	< 500
C3 Complement (mg/dL)	45	82–193
C4 Complement (mg/dL)	< 3	16–48
DsDNA titer	1:2560	Negative
CRP (mg/dL)	7.83	< 0.50

Electrocardiography showed sinus tachycardia with low QRS voltage in the precordial leads. Neither ST-segment elevation nor PR-interval depression was demonstrated. Chest radiography was unremarkable. Echocardiography was performed, which revealed a small pericardial effusion and a mildly dilated RV with reduced systolic function and severe hypokinesis of the basal-to-mid free wall consistent with McConnell’s sign (Videos 1, 2).

**Video 1:** Two-dimensional TTE, apical RV focused view The initial TTE demonstrated a dilated right ventricle with severe hypokinesis of the basal-to-mid free wall and preserved contractility of the apex consistent with McConnell's sign. TTE: Transthoracic echocardiogram; RV: right ventricular

**Video 2:** Two-dimensional TTE, apical RV focused view with contrast enhancement Contrast enhancement again demonstrated a dilated right ventricle with severe hypokinesis of the basal-to-mid free wall and preserved contractility of the apex consistent with McConnell's sign. TTE: Transthoracic echocardiogram; RV: right ventricular

Traditional indices of RV systolic function were discordant, with tricuspid annular plane systolic excursion (TAPSE) abnormal at 1.5 cm and tissue Doppler (S’) normal at 12.5 cm/s (Figures 1, 2). RV fractional area change was abnormal at 30%. RV systolic pressure was only mildly elevated and estimated at 30-34 mmHg by tricuspid valve regurgitation evaluation (Figure 3). Global RV longitudinal strain was significantly abnormal at -7.8%, with significant dysfunction of the basal and mid RV free wall (Video 3, Figure 4).

**Figure 1:**
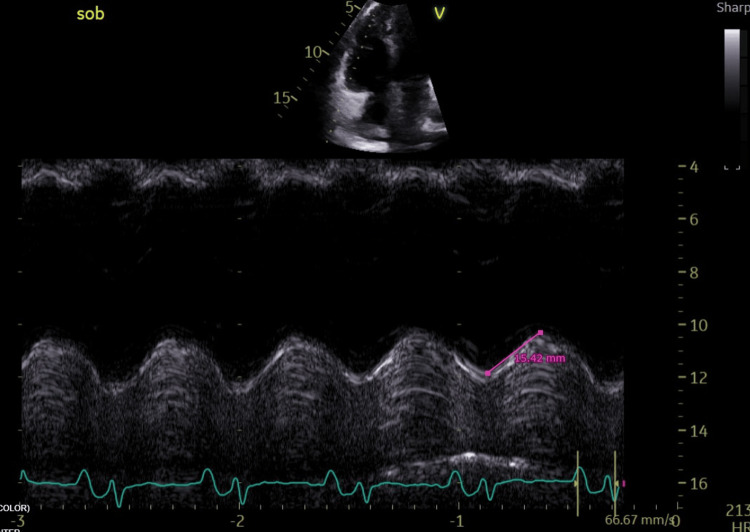
TTE M-mode, apical RV focused view through the tricuspid valve annulus M-mode demonstrated decreased tricuspid annular plane systolic excursion (TAPSE) of 1.5 cm. TTE: Transthoracic echocardiogram; RV: right ventricular

**Figure 2:**
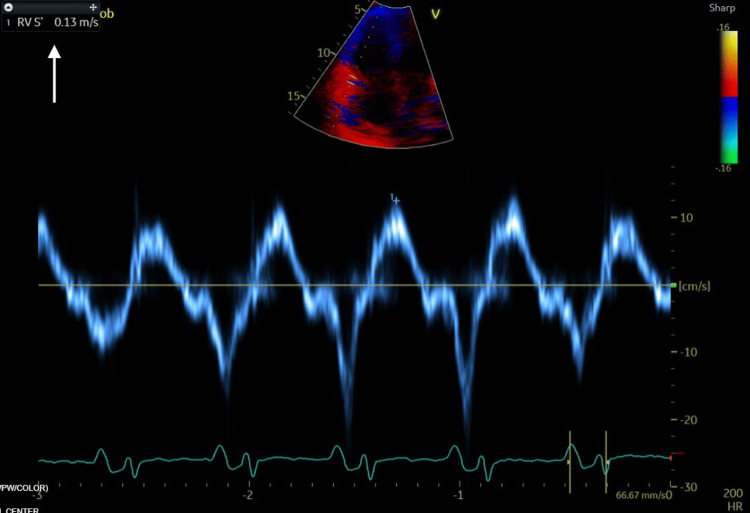
TTE tissue Doppler imaging, apical RV focused view focused on the tricuspid annulus Tissue Doppler evaluation demonstrated a normal tissue Doppler (S’) velocity of 12.5 cm/s. TTE: Transthoracic echocardiogram; RV: right ventricular

**Figure 3:**
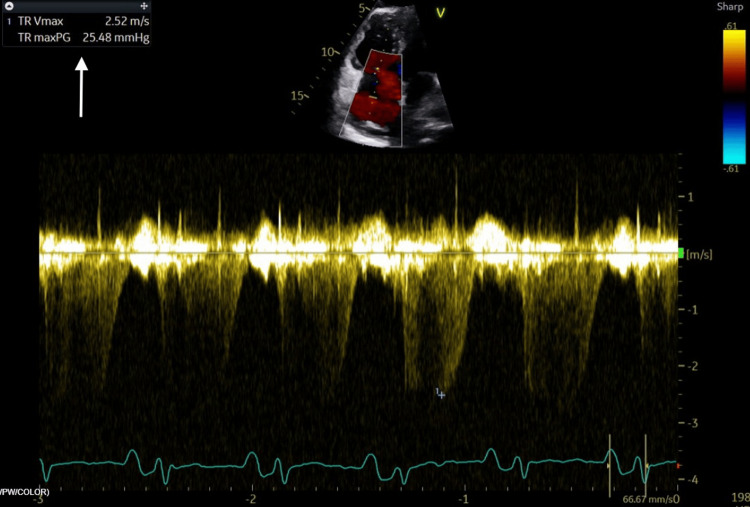
TTE continuous wave Doppler, apical RV focused view through the tricuspid valve Continuous wave Doppler evaluation demonstrated a tricuspid regurgitation velocity of 2.5 m/s. Inferior vena cava evaluation suggested a right atrial pressure of 5-10 mmHg, resulting in an estimated right ventricular systolic pressure of 30-35 mmHg. TTE: Transthoracic echocardiogram; RV: right ventricular

**Video 3:** TTE speckle-tracking echocardiography, apical RV focused view Speckle-tracking strain analysis demonstrated an abnormal right ventricular global longitudinal strain of –7.8%. TTE: Transthoracic echocardiogram; RV: right ventricular

**Figure 4:**
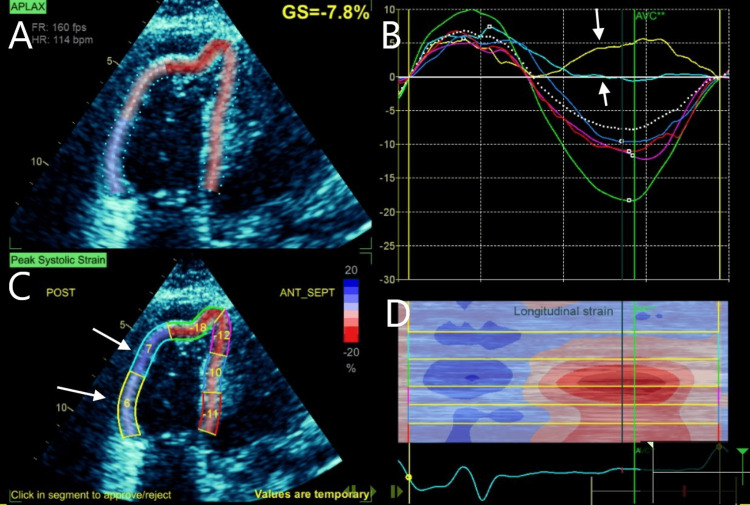
TTE speckle- tracking echocardiography, apical RV focused view Speckle-tracking strain analysis demonstrated abnormal global longitudinal strain, driven predominantly by reduced basal and mid free wall strain. The basal and mid free wall segments (blue and yellow) have positive values (panel C) and abnormal strain curves (panel B), consistent with akinesis and dyskinesis with systole. TTE: Transthoracic echocardiogram; RV: right ventricular

Given the elevated D-dimer and echocardiographic findings, acute PE was strongly suspected, and anticoagulation with unfractionated heparin was immediately initiated. Urgent chest computed tomography with intravenous contrast was performed, which was negative for PE or evidence of pulmonary fibrosis but revealed a small pericardial effusion (Figure 5, Video 4). Bilateral lower extremity venous Doppler examination was performed and was negative for deep venous thrombosis. Acute myocardial infarction with RV involvement was considered; however, ischemic evaluation was deferred given the lack of electrocardiographic findings concerning for ischemia and normal left ventricular function. Myocarditis was strongly suspected, and a cardiovascular magnetic resonance (CMR) imaging at 1.5 Tesla was performed to help further establish a diagnosis. Unfortunately, the images were suboptimal due to patient movement and difficulty with breath holds, and the myocardium was poorly visualized. Despite the challenges with image acquisition, a small to moderate pericardial effusion was again seen.

**Figure 5:**
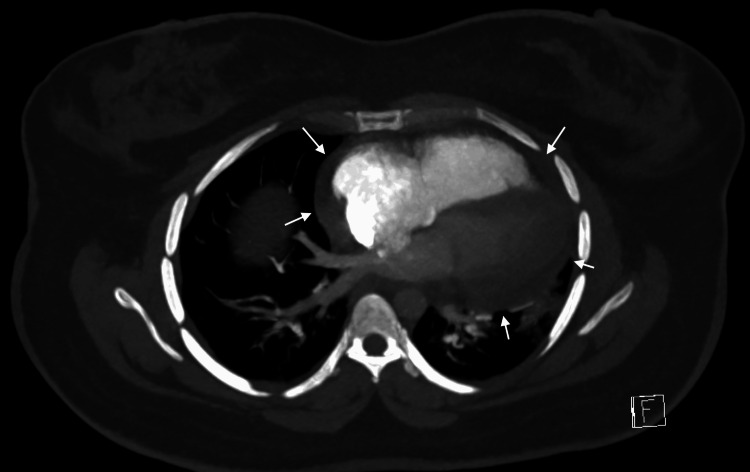
Computed tomography with intravenous contrast, axial view through the thorax CT demonstrated a small circumferential pericardial effusion.

**Video 4:** Computed tomography of the chest with intravenous contrast, axial display from the upper thorax to the abdomen The CT scan demonstrates the presence of pericardial effusion and the absence of pulmonary embolism.

Based on her pericardial findings and elevated troponin, a presumed diagnosis of myopericarditis associated with a concomitant SLE flare was made. The patient was initiated on anti-inflammatory therapy with prednisone 60 mg by mouth daily in addition to their home hydroxychloroquine to treat the SLE flare and colchicine 0.6 mg by mouth twice daily for myopericarditis. The chest pain and dyspnea improved over the following days, and follow-up echocardiography after four days of therapy revealed resolution of the RV dysfunction, with normal chamber size and wall motion. Given the improvement in symptoms, the patient was discharged home with a prolonged course of oral anti-inflammatory therapy.

The patient was evaluated one month after discharge and reported continued improvement in their pleuritic chest pain and a gradual decrease in her joint swelling. Echocardiography showed normal RV size and function without regional wall motion abnormalities (Video 5). TAPSE was improved to 2.1 cm and RV S’ improved to 14.0 cm/s (Figures 6, 7).

Video 5: Two-dimensional TTE, apical RV focused view

Follow-up TTE one month later demonstrated an RV of normal size and function, with no residual wall motion abnormalities. 

**Figure 6:**
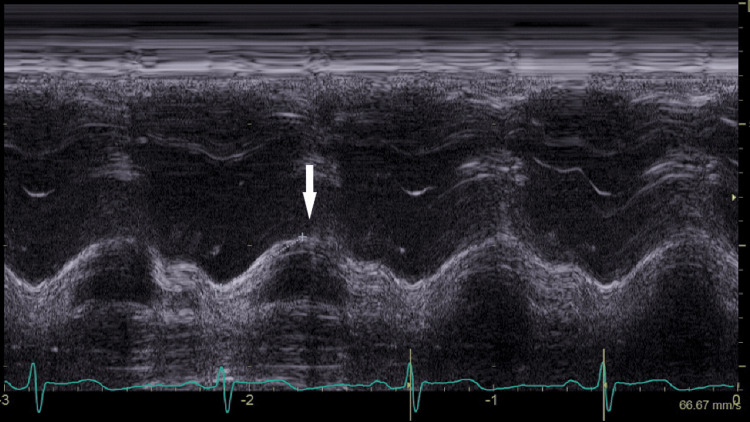
TTE M-mode, apical RV focused view through the tricuspid valve annulus Follow-up evaluation of RV function demonstrated improved tricuspid annular plane systolic excursion (TAPSE) of 2.1 cm.

**Figure 7:**
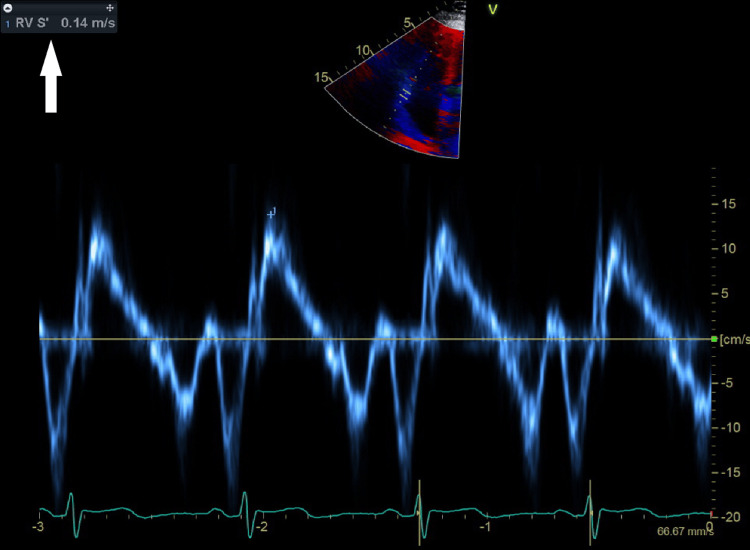
TTE tissue doppler imaging, apical RV focused view focused on the tricuspid annulus Follow-up evaluation of RV function demonstrated normal tissue doppler (S’) velocity of 14.0 cm/s.

## Discussion

McConnell’s sign is an echocardiographic finding most commonly associated with acute PE in the literature. While first described as having a sensitivity of 77% and a specificity of 94% for acute PE, the sensitivity of the sign has been reported to be significantly lower in more recent reviews. A meta-analysis of 21 studies by Fields et al. showed that McConnell’s sign has a sensitivity for acute PE of only 22%, but still with a high specificity of 97% [2]. Despite a consistently high specificity and positive predictive value for the diagnosis of acute PE, McConnell’s sign has been described in other disease states. Pulmonary hypertension, acute myocardial infarction, coronary air embolus, and acute chest syndrome have all been associated with McConnell’s sign on echocardiography [4,5].

This case represents a rare and not well-recognized presentation of McConnell’s sign in association with myopericarditis and SLE flare. To arrive at this diagnosis, other potential causes of McConnell’s sign were evaluated and excluded during the patient’s hospital admission. Acute PE and deep venous thrombosis were ruled out with computed tomography and lower extremity venous Doppler evaluation, and underlying pulmonary hypertension was not found on echocardiography. Myocardial infarction with RV involvement was considered but believed to be unlikely given the absence of ischemic changes on electrocardiography and normal left ventricular function without wall motion abnormalities on echocardiography. CMR imaging was notable for pericardial inflammation without definitive myocardial involvement, a finding that may be attributable to overall suboptimal image quality. The degree of troponin elevation suggests myocardial injury despite CMR imaging. The diagnosis of myopericarditis was further supported by a clinical presentation consistent with an inflammatory process - evidenced by the SLE flare, pleuritic chest pain, and the presence of a pericardial effusion.

The mechanism of McConnell’s sign seen in this case is not known but is theorized to be due to direct RV free wall injury from myocarditis. Modern strain analysis has shown the entire RV free wall (including the apex) is dysfunctional in McConnell’s sign in the setting of PE [6,7]. In our case, the patient had abnormal basal and mid RV free wall speckle-tracking strain but preserved RV apical strain, suggesting a focal process limited to the basal and mid segments, likely due to myocarditis.

Myocarditis and pericarditis are inflammatory disorders of the heart and pericardium that have traditionally been considered distinct clinical entities. Myocarditis classically presents with symptoms of heart failure, elevated troponin, abnormal wall motion on echocardiography, and arrhythmias, whereas pericarditis is typically associated with pleuritic chest pain, electrocardiographic changes (diffuse ST-segment elevation or PR-interval depression), a friction rub, and pericardial effusion. In practice, patients frequently present with overlapping features, prompting a paradigm shift in recent years towards understanding myocarditis and pericarditis as part of a spectrum of disease. The ACC has recently developed two expert consensus documents on the diagnosis and management of pericarditis and myocarditis [8,9]. They emphasize a stage-based classification of myocarditis based on disease severity and the presence of markers of myocardial injury and inflammation. By contrast, the ESC published its updated guideline document in 2025, which defines myocarditis in terms of tiered certainty [10]. They recommend classifying both myocarditis and pericarditis as definite, possible, or unlikely based on clinical features and cardiac resonance imaging and endomyocardial biopsy findings. The recent ESC guidelines also introduced the term 'inflammatory myopericardial syndrome' as a broad term during the initial diagnostic phase until a final diagnosis is made.

Cardiovascular magnetic resonance imaging has emerged as a mainstay in the diagnosis of myocarditis, specifically, with endomyocardial biopsy increasingly reserved for intermediate or high-risk cases. When patients present with overlapping features, a diagnosis of perimyocarditis (predominant pericarditis) or myopericarditis (predominant myocarditis) can be made [10].

Left ventricular dysfunction with regional or global wall motion abnormalities is a frequent complication of myocarditis, but combined biventricular or isolated RV dysfunction can also occur. Abnormal RV speckle-tracking strain has been reported in myocarditis with preserved left ventricular function, suggesting that subclinical RV dysfunction can occur without overt wall motion abnormalities [11]. Isolated RV myocarditis with associated systolic dysfunction has been described in case reports; however, only ventricular dilation and global hypokinesis have been reported [12-15]. Regional wall motion abnormalities have not been previously described in inflammatory myopericardial syndromes. One hypothesis is that isolated RV basal and mid free wall myocardial inflammation may lead to hypokinesis and mimic the findings of McConnell’s sign in PE, but further study is needed.

## Conclusions

McConnell’s sign is an echocardiographic finding most often encountered in the context of acute PE. While having a relatively high specificity for acute PE, other causes have been described in the literature. This report describes a distinct regional RV wall motion pattern in an inflammatory myopericardial syndrome with concomitant SLE flare, which challenges the traditional diagnostic specificity of McConnell’s sign. While RV dysfunction and dilation have been reported in myocarditis, including subclinical or isolated RV involvement, acute regional wall motion patterns such as McConnell’s sign have not been previously described. Additionally, this case also highlights the value of speckle-tracking strain analysis in refining the differential diagnosis and determining the underlying mechanism of McConnell’s sign.
